# Energy Versus Throughput Optimisation for Machine-to-Machine Communication

**DOI:** 10.3390/s20154122

**Published:** 2020-07-24

**Authors:** Emma Fitzgerald, Michał Pióro, Artur Tomaszewski

**Affiliations:** 1 Department of Electrical and Information Technology, Lund University, 221 00 Lund, Sweden; emma.fitzgerald@eit.lth.se; 2 Institute of Telecommunications, Warsaw University of Technology, 00-665 Warsaw, Poland; a.tomaszewski@tele.pw.edu.pl

**Keywords:** wireless sensor networks, multicast, transmission scheduling, integer programming, IoT, machine-to-machine communication

## Abstract

We investigate the trade-off between energy usage and (packet) throughput in wireless mesh networks performing machine-to-machine communication. For this we provide a novel mixed-integer programming formulation to maximise the throughput while maintaining minimal energy usage, together with an effective price-and-branch solution algorithm based on column generation. The resulting optimisation model is the main original contribution of the presented paper. We conducted a numerical study using network examples from 10 to 40 nodes, in which periodic sensor measurements are aggregated and disseminated to actuators. In almost all cases, we were able to achieve maximum throughput and minimum energy usage simultaneously, and in those cases where this was not possible, the costs incurred in one objective in order to achieve the other were typically low. The solution times for all network sizes were of the order of seconds, showing that our optimisation model is feasible to use in practice.

## 1. Introduction

In machine-to-machine communication, both sensors and actuators may be present in the network and communicating with each other. Measurements are taken by sensor nodes, and this data must then be transmitted through the network to actuator nodes that make use of it in order to take decisions or set control parameters. Sensor data may be processed in the network, with data aggregation being a typical case of such processing, encompassing simple operations such as counting the number of events observed, up to more sophisticated functions such as data compression. Once aggregated, the data may then be disseminated to multiple actuators via multicast transmissions, to which wireless mesh networks are particularly suited due to the broadcast nature of the wireless medium.

In our previous work [1,2], we addressed the problem of determining optimal (data) routing configurations for data aggregation and dissemination in order to minimise the energy usage of the network spent on packet aggregation and transmission. These configurations specify routing of sensor measurements to actuators, as well as which intermediate nodes should aggregate and broadcast data. We considered two types of energy optimisation: minimisation of the total energy usage of all nodes in the network, and min-max energy usage, in which the energy costs are balanced amongst the different nodes. We now build on that work in order to optimise the throughput for machine-to-machine communication in wireless mesh networks, as well as to explore the trade-offs between energy usage and the throughput in question.

In the case of periodic sensor measurements that we consider in this paper, throughput maximisation may be a goal in itself, or may be desirable to free up resources for other network applications. The throughput also has an important secondary effect on the overall energy usage. This is because once all sensor measurements have been processed and transmitted, nodes may be able to sleep, thus saving energy. In [1,2], we considered only the energy required for aggregation and data transmission; however, the energy used while the node is idle but awake is also in many cases significant, and thus should be minimised for a complete, energy-efficient solution.

In this paper, we adopt the approach from the work in [3] for throughput maximisation of periodic multicast traffic but extend it to take into account the energy usage. Specifically, we develop optimisation formulations to maximise the throughput of sensor measurements reaching actuator destinations under the requirement that the energy usage is not increased above a given threshold. We conducted a numerical study to test the performance of our optimisation models, and to investigate the trade-off between energy usage and throughput. Our results show that in most cases, it is possible to simultaneously achieve the maximum throughput and minimum energy usage, and even in cases where this is not possible, the additional energy cost needed to achieve the maximum throughput is low. This, in fact, is a non-obvious observation.

The main novelty of the presented work consists in introducing an original approach to optimisation of data aggregation and transmission in wireless mesh networks applying multicast packet transfer. The approach considerably extends our previous work described in [1,2,3].

The rest of this paper is organised as follows. In Section 2, we discuss related work. Section 3 describes our problem setting and its motivation. Section 4 and Section 5 then give our optimisation formulation and the algorithm used to solve it. In Section 6, we present the results of our numerical study, and finally in Section 7 we conclude this paper.

## 2. Related Work

Energy saving in wireless mesh networks, including wireless sensor networks, is an important issue closely related to network lifetime maximisation. There is a variety of papers in this field, see, for example, in [4,5,6], but most of them are not intended to consider traffic throughput efficiency and data aggregation in the precise way, as we do in this paper. Those papers (and others) are discussed in [2].

While data aggregation in wireless mesh networks has been studied extensively for the past two decades [7], in most of that previous work the application scenario considered is that of a sensor network collecting measurements to be sent to a single sink. Popular protocols that cater to this use case include LEACH [8] and PEGASIS [9], with much other work based on these. Recently, the focus has shifted away from this traditional data gathering model towards other goals, for example, aiming to minimise latency [10,11,12,13,14].

However, aggregation for machine-to-machine communication remains an open challenge, particularly in combination with energy efficiency [15]. In this area, use cases in which data is not only aggregated, but also disseminated to multiple destinations, require particular consideration. This is first due to the new challenges this causes in routing of sensor measurements, which we addressed in [1], and also because this involves multicast transmissions. Research considering multiple data sinks is limited, but includes the works in [16,17,18]. However, none of these works addresses multi-hop routing with multicast transmission, which we focus on in this paper.

Multicast transmission has been studied in the context of wireless networks for some time, however mostly in the context of devising efficient algorithms or protocols, rather than optimisation. The work in [19] examines the differences between wired and wireless networks in the context of multicast, and evaluates different algorithms for construction of broadcast and multicast trees in terms of energy efficiency. The work in [20] gives a protocol for multicast routing in ad hoc wireless networks, and in [21], the capacity of large multi-hop wireless networks employing multicast transmission is studied.

In more recent work, multi-hop networks are used as the setting in [22], where a two-stage approach is taken to first construct a multicast routing tree, and then determine the minimum transmission power for each node in the tree, with the aim of minimising energy usage. The authors of [23] also study energy-efficient multicast routing in multi-hop wireless networks, but using a cooperative approach based on amplify-and-forward, and focusing on smart medical applications. The authors of [24] consider multicast routing in delay-constrained networks, again with the goal of energy efficiency, taking into account the energy required for both transmission and reception. In these works, however, throughput is not considered as a performance goal. While we aim for energy efficiency, as do the works mentioned above, we aim to also maximise the throughput, and to investigate what gains are possible in the throughput when relaxing the minimum energy requirement, and, vice versa, how much energy can be saved by reducing the throughput from the maximum. Moreover, we focus on networks carrying out machine-to-machine communication with data aggregation, which calls for a specific traffic model suitable to this application.

In [25], in addition to energy efficiency, throughput maximisation is also considered. However, there throughput refers to the average throughput achievable by each individual node in the network. In our paper, we instead consider the end-to-end throughput actually provided to the data streams, which depends not only on the achievable data rate at each node, but also on the scheduling of transmissions, taking into account the specific characteristics of multicast transmission. For multicast, the broadcast nature of radio communications becomes important as then a packet broadcast from a given node can simultaneously reach several of its intended destinations. However, with broadcast it is possible for the transmitted packet to be successfully received by some of its intended destinations, while at others there is too much interference for the packet to be decoded.

Thus, in order to optimise transmission scheduling, this phenomenon must be taken into account, and so approaches used for unicast traffic (see, for example, in [26] and the references therein) are no longer suitable. Appropriate extensions are not straightforward and require a substantial modification of the optimisation model for packet transmission scheduling. Such a model, suitable for our aims, was recently presented in [3]. However, in [3], energy was not considered and the goal was solely throughput maximisation. In this work, we extend the model from the work in [3] to take into account the energy used by the nodes. Using the modified model, we then investigate the trade-off between the two goals of energy minimisation and throughput maximisation in our numerical study.

## 3. Problem Setting

In this section, we will detail the problem setting we consider. We first discuss applications that motivate our work. After that, we give a general characterisation of the optimisation problem considered in the paper, followed by a detailed description of its two main subproblems.

### 3.1. Motivating Applications

In the paper we consider wireless mesh networks (WMN), in particular, wireless sensor and actuator networks (WSAN), supporting a machine-to-machine communication application. Within the network are sensor nodes that collect measurements and destination nodes that use these measurements, which could be either actuators needing the measurement data to take actions or set control parameters, or gateways collecting measurement data for analysis or other purposes. In general, the measurements from a given sensor may be used by multiple actuators, and similarly, each actuator can use measurements from multiple sensors. Moreover, while transiting through the network, sensor measurements can be aggregated by intermediate nodes along the way.

A large class of applications that match the above description are control applications. There, aggregation amounts to executing a control algorithm on sensor data (from possibly multiple sensors) to derive the controller output value. The output value is delivered to a respective actuator, so that it can drive some physical quantity in a desirable way. It is common that, just as in our description, each actuator’s control algorithm is driven by readings from the actuator’s specific subset of the sensor nodes. If actuators drive different physical quantities, the control algorithms, and thus the inputs they take, are different. Even if two actuators drive the same quantity, their control algorithms most likely use sensor readings from an actuator-specific zone of the space being controlled. Furthermore, clearly, readings from a single sensor may be used as input to the control algorithms of multiple actuators, especially if the actuators drive different physical quantities.

The architectures of WSAN-based control applications are usually classified as automated and semi-automated ones [27]. In the former, the control algorithms are executed in the WSAN itself, while in the latter, sensor data are transmitted to sinks and the control algorithms are executed outside of the WSAN (e.g., in the cloud). We adopt the automated architecture. Moreover, we do not assume that the control algorithm for some specific actuator is pinned to the actuator’s node itself. The execution of the actuator-specific control algorithm (which we call aggregation) may occur at an intermediate node or may even be distributed among a number of intermediate nodes, as dictated by availability of input sensor readings.

WSAN-based control applications are used in many domains, including smart buildings/ cities  [28,29] and precision agriculture/smart farming [30,31]. For example, in the smart building case, one can use readings from light intensity, occupancy, temperature, humidity and CO2 sensors to drive lighting, shades, thermostat settings as well as opening and closing windows, in a per-room manner. A control algorithm for a lighting actuator (switch) could use readings from the light intensity and occupancy sensors. A control algorithm for a window actuator could use readings from the sensors reporting occupancy, CO2 level as well as inside and outside temperature and humidity. In the precision agriculture case, the application can monitor plants’ environmental conditions, like soil and ambient temperature, irrigation water and soil conductivity, soil and irrigation water PH, nutrient composition data and irrigation water properties, and then can actuate to maintain optimal values of those quantities. (Other applications here are automatic traffic control (actuator = traffic light), fire detection (actuator = alarm) and wastewater monitoring (actuator = wastewater sampling device) in the smart city, and automatic irrigation control (actuator = irrigation valve) in smart farming.)

### 3.2. General Description of the Problem

In general, the problem we consider is to design a sensor network in which sensor nodes collect measurement data and send that data to destination nodes, which might be, for example, actuators or network gateways. The data is collected periodically in measurement cycles of certain duration. In every measurement cycle, the data collected by each sensor node is sent to one or more destination nodes via a sequence of transit nodes, with each transit node aggregating data received from its multiple neighbours and using multicast transmission while sending the aggregated data. The design problem consists of optimising the routing of the measurement data, and optimising the scheduling of node transmissions. The objective of the design might be to minimise the energy usage, which maximises the network lifetime, or to minimise the length of the measurement cycle, which maximises the measurement data throughput (i.e., how much measurement data can be collected and sent in a given time).

While combining the two subproblems leads to a complex optimisation model, in [1] we consider only the first subproblem—we design the routing of measurement data, i.e., routing configuration, by deciding to which neighbours the node sends its aggregated data. Our objective is to minimise the energy consumed by the node, which we assume consists of the energy required for data aggregation and the energy required for data reception and transmission. While not designing transmission schedule, we make a simplifying assumption that each node transmits data at most once, i.e., it sends the aggregated data to all required neighbours in a single multicast transmission, and thus the energy it uses for transmission is minimal.

In the current paper we go further, by considering the second subproblem—we design a transmission schedule and optimise packet transmissions by deciding on the number of timeslots of the measurement cycle required for transmissions, and designing the set of collision-free transmissions in each timeslot: we decide on the set of nodes that transmit in the timeslot, and for each transmitting node—on the set of the node’s neighbours that receive its data. We assume that routing configuration is given as a result of solving the first subproblem: the routing configuration defines to which neighbours the node must send its aggregated data. Our objective is to minimise the number of required timeslots of the measurement cycle, and thus to maximise the measurement data throughput. For that we extended the optimisation model introduced in [3].

One can notice that the number of required timeslots is upper bounded by the number of nodes—we can make each node transmit in a separate timeslot, using a single multicast transmission to reach all its required neighbours. However, the number of timeslots might be decreased if a number of parallel transmissions are designed for a single timeslot. The question is, however, if while parallelising transmissions we can still make each node transmit only once, and therefore use the minimal energy required for transmission. Thus, we validate the assumption from the first subproblem that each node transmits only once, and examine the potential trade-off between the number of timeslots of the measurement cycle and the energy used by network nodes for transmissions.

### 3.3. Routing Configuration Optimisation

**Figure 1:**
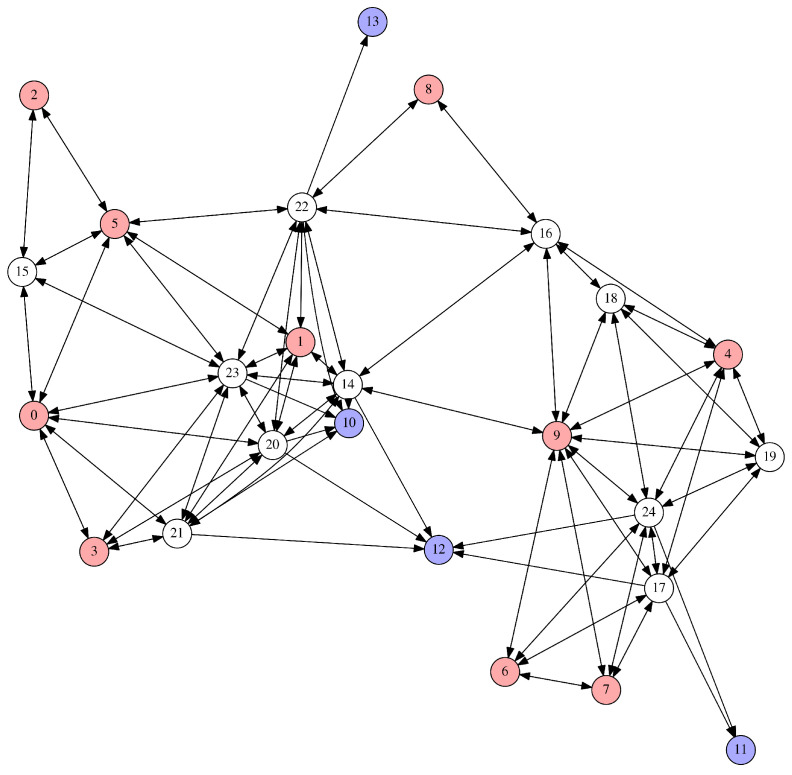
An example network of the type we consider in this paper. Nodes shown in red are origin (sensor) nodes that first of all produce sensor measurements, but also transit and aggregate data. Nodes shown in white are aggregator nodes, that both transit and aggregate data on its way through the network. Nodes shown in blue are destination nodes for the multicast data streams originating at the sensor nodes.

In our considered scenario (whose notation is summarised in Table 1), the set of nodes V is composed of three mutually disjoint subsets: the set of origin (sensor) nodes O, the set of aggregator nodes N and the set of destination nodes D. Thus, V=O∪N∪D. By assumption, origin nodes generate, transit and aggregate packets; aggregator nodes transit and aggregate packets; and destination nodes aggregate packets but do not transit them. An example network consisting of 25 nodes is shown in Figure 1, with origin nodes shown in red, aggregator nodes shown in white and destination nodes shown in blue.

In each measurement cycle a set of measurements is collected at the origin nodes to be delivered to the destination nodes. Each destination node d∈D receives a set of (possibly aggregated) measurements from at least *K* origin nodes, where K≤|O| is a given parameter. Let O(d) (where O(d)⊆O and |O(d)|≥K) denote the set of origins whose measurements should reach destination d∈D. Each packet containing the measurement from origin o∈O(d) follows a path P(o,d) from *o* to *d* that traverses a subset, denoted by V(o,d), of transit nodes in (O\{o})∪N. Such a packet is aggregated with other packets sent over paths $P(o^{\prime },d^{\prime })$ of other origin–destination pairs $\left(o^{\prime },d^{\prime }\right)$. The aggregation in question is performed for all packets (possibly themselves aggregates) containing measurements from different origins that arrive at the same aggregator node. We thus assume that measurements are aggregated whenever possible. Moreover, when any two origin–destination paths meet for the first time, at node v(1), say, they traverse a common sequence of nodes, sequence v(1),v(2),…,v(n), say, and when they split at node v(n) they do not meet again. This avoids any possibility for duplicate measurements arriving at the same destination node, i.e., it prevents the nodes from receiving the same measurement on more than one incoming arc. (See Section 6 in [1] for details of the aggregation model.)

Using the optimisation models described in [1], we can obtain a routing configuration—the set of paths that sensor measurements take to reach their destinations, as well as the nodes at which aggregation occurs—that uses the minimum energy to deliver all packets, taking into account energy costs for both aggregation and transmission operations. Under these models, energy can be minimised in one of two ways: either the total energy used by all nodes in the network is minimised, or the maximum energy used by any node in the network is minimised (known as min-max optimisation). The models take into account the energy used for aggregation of measurements, as well as the energy used for transmissions. Observe that these two energy consumption factors are contradictory: if the number of (transit) nodes increases, then less energy is spent on aggregation while more is spent on data transmission, and vice versa.

Note that energy required for signalling or other overheads to set up the routing configuration is not included. This signalling will be different depending on the particular network used: whether it has a connection to the Internet or is standalone, whether only one configuration is used throughout the lifetime of the network or the configuration can be changed during operation [2] and whether the configuration can be preprogrammed before deployment or the network is self-configuring. These are complex issues in themselves, and so here we focus on the operation of a single configuration, and exclude signalling and overhead so as to keep the results general to any specific protocol used.

In the remaining part of this paper, we will assume that we already have such a routing configuration and we will then seek to maximise the throughput of the sensor measurements under the constraint that the energy used should remain at the already determined minimum value. The configuration in question is the subgraph of the original network graph generated by the set of nodes of (optimised) paths P(o,d),d∈D,o∈O(d) (note that the sets O(d) are optimised as well). It follows that the sets of origin nodes $O^{\prime }$, aggregate nodes $N^{\prime }$ and destinations nodes $D^{\prime }$ of this subgraph are equal to, respectively, ${⋃}_{d\in D}O\left(d\right)$, $\left({⋃}_{d\in D,o\in O(d)}V\left(o,d\right)\right)\backslash O^{\prime }$, and D.

Let $B:=O^{\prime }\cup N^{\prime }$. As the destination nodes in D do not forward packets, only the nodes in B broadcast packets. In fact, each of them broadcasts only one packet for each set of measurements collected, as if a node receives multiple packets, it will aggregate them. Note that for each v∈B, the assumed set of paths uniquely determines the set of nodes R(v) that directly receive the packet sent from *v*. By construction, all nodes in R(v) are in transmission range of node *v*, as this is assumed for the original network. Thus, essentially only one broadcast is sufficient to deliver the packet from any v∈B to all its destinations in R(v), barring any interference.

### 3.4. Frame Length Minimisation

Having specified a routing configuration, we proceed to describing the issue of packet transmission scheduling optimisation, which is central to this paper. It is important to note that the routing configuration optimisation described above does not consider the cyclicity of sensor measurements: it simply requires that a given set of measurements be delivered to the required destinations with a minimum energy consumption. Moreover, it assumes that the part of energy consumed by packet transfer (the second part is energy consumed by measurement aggregation) is the energy spent on packet transmissions that is simply proportional to the number of broadcasting nodes |B| (thus, it is assumed that each node transmits only once), plus the energy spent on packet receptions that is proportional to the total number of active links $\sum_{v\in B}\left|R\left(v\right)\right|$.

However, to avoid collisions packet transfers must be scheduled in a number of consecutive time slots (the time slot is supposed to be the time required to transmit the packet over a radio link). Due to the cyclicity of measurements assumed in the network setting studied in the current paper, those time slots must be grouped into the frame that is going to be repeated periodically. The length of this frame, i.e., the number of its time slots, must now be optimised, as it defines the minimum length of the measurement cycle. Moreover, the shorter the measurement cycle, the higher the network throughput, while at the same time, as will soon become clear, the shorter the measurement cycle, the higher the energy spent on packet transfer (in particular, that energy might not be proportional to |B|). As already mentioned, apart from finding the transmission pattern of the frame, the current paper is devoted to analysing and solving this particular trade-off.

The transmission pattern of the frame is specified by the transmission pattern of each time slot; the latter is defined in the form of a *compatible set* (c-set in short) [3]. A c-set is described by a set of (simultaneously) broadcasting nodes W and, for each v∈W, the set of nodes U(v) receiving the signal from *v*. Certainly, such an arrangement is valid if every node in U(v) is able to decode the signal from *v*, i.e., the nodes receiving from *v* are not interfered with by the signals that are simultaneously broadcast from the remaining nodes in W, i.e., from nodes in W\{v}.

For optimisation purposes, c-sets are identified by feasible solutions of the system of linear inequalities imposed on binary variables $X_{v}$ (v∈B) and $Y_{vu}$ (v∈B,u∈R(v)):

(1a)Xv≥Yvu,v∈B,u∈R(v)(1b)Xv≤∑u∈R(v)Yvu,v∈B(1c)Xv+∑u∈{w∈B:R(w)∋v}Yuv≤1,v∈B(1d)p(v,u)+M(1−Yvu)≥γη+∑w∈B\{v,u}p(w,u)Xw,v∈B,u∈R(v)(1e)Yvu∈B,v∈B,u∈R(v)(1f)Xv∈B,v∈B.

Above, p(v,w) is the transmission power received at node *w* from node *v*, η is the noise power, and γ>1 is the assumed signal-to-interference-to-noise ratio (SINR) threshold (for more detailed explanation of the transmission parameters see in [26]). Constraints (1a) and (1b) force the node to broadcast ($X_{v}=1$) if its broadcast signal is to be received by any of its neighbours ($Y_{vu}=1$); otherwise, $X_{v}$ is forced to be zero. At the same time, these two constraints assure that when node *v* broadcasts then at least one of its destination nodes u∈R(v) receives its signal. Constraint (1c) ensures that if a node is transmitting then it cannot receive, and if a node is not transmitting then it can be receiving, but from at most only one neighbouring node.

Finally, constraint (1d) expresses the SINR condition. Note that the second term on the left-hand side of (1d) is added to cancel this constraint (using a “big M” constant *M*) whenever $Y_{vu}=0$. *M* can for example be defined as

(2) $$M\left(v,u\right):=\gamma (\eta +\sum_{w\in B\backslash \{v,u\}}p\left(w,u\right)),$$

i.e., the upper bound on the right-hand side of (1d).

Any solution of system (1) defines the corresponding c-set as follows, $W:=\{v\in B:\;X_{v}=1\}$ and $U\left(v\right):=\{u\in R\left(v\right):\;Y_{vu}=1\}$. Below, for a given network we will denote the family of all such c-sets by $\hat{C}$ and its elements by *c*. For a given $c\in \hat{C}$, the set of broadcasting nodes will be denoted by W(c), and the sets of receiving nodes by U(c,v),v∈W(c).

The above constraints admit trivial solutions, in which, for example, no nodes transmit or receive. However, as we will generate c-sets using an optimisation problem, this will not cause any issues. The c-set that will be generated each time will be the one that gives the optimal value of the provided objective function, related to improving the throughput of the network. As any trivial solutions will not improve the throughput, such c-sets will never be generated, and it is not necessary to include additional constraints to exclude them.

Note that for a given set B of broadcasting nodes and the corresponding sets of receiving nodes R(v),v∈B, all the required broadcasts can be realised in a frame consisting of |B| slots, each applying a c-set c(v) with W(c(v))={v} and U(c(v),v)=R(v). The family of all such c-sets, i.e., the subfamily {c(v):v∈B} of $\hat{C}$, will be denoted with C(B). However, although such a frame is optimal in terms of transmission energy, it could be unnecessarily long. If we instead apply c-sets with more than one broadcasting node, we can potentially reduce the frame length, although we will also add interference, which in general may necessitate more than one transmission for a given broadcasting node to reach all receivers.

This observation leads to the optimisation problem studied in this paper, called the *frame minimisation problem* (FMP): find the minimum frame length *T*, together with a c-set $c\left(t\right)\in \hat{C}$ for each time slot t=1,2,…,T, such that $R\left(v\right)\subseteq {⋃}_{t\in T:\;v\in W(c(t))}U\left(c\left(t\right),v\right)$ for each v∈B, under the transmission energy constraint that keeps the total number of node broadcasts in the frame below a given limit. FMP will be defined in the next section. Although an individual measurement may traverse the network over multiple frames, in each frame (after an initial warm-up period), one set of measurements is delivered to the destinations. Minimising the frame length thus maximises the throughput of the network.

## 4. Problem Formulation

Let us consider a fixed and given routing configuration with the set of broadcasting nodes B and the sets of receiving nodes R(v),v∈B. Assume also that the energy used for broadcasting a packet is the same for each broadcasting node, and is normalised to one energy unit. The following mixed-integer programming (MIP) formulation specifies the frame minimisation problem for a given subfamily C of the family of all c-sets $\hat{C}$ and a given integer parameter Δ≥0 specifying the energy margin:

(3a)FMP(C,Δ):minT=∑c∈CTc(3b)[λvu≥0]∑c∈C(v,u)hvc≥1,v∈B,u∈R(v)(3c)[πcv≥0]hvc≤Tc,v∈B,c∈C(v)(3d)[θ≥0]∑v∈B∑c∈C(v)hvc≤|B|+Δ(3e)hvc∈B,v∈B,c∈C(v)(3f)Tc∈R,c∈C.

Above, the quantities in square brackets on the left-hand sides of the constraints are the corresponding dual variables to be used in Section 5 for the linear relaxation of FMP (C); the notation used in (3) is summarised in Table 2.

Each binary variable $h_{vc}$ (defined for a given broadcasting node *v*, and a given c-set *c* that can be used to broadcast the packet generated at *v*, see (3e)) is equal to 1 if, and only if, such a broadcast is actually scheduled in the frame. Therefore, the inequality in constraint (3b) assures that during the frame the packet in question will reach each receiver u∈R(v). In order to ensure that the required c-sets are applied in the frame, binary variables $T_{c},\;c\in C,$ are used. For any given *c*, this is achieved due to constraints (3c) considered for all nodes broadcasting in *c*, i.e., for all v∈W(c). These constraints force $T_{c}$ to be equal to 1 when at least one $h_{vc}$ is equal to 1, i.e., when the considered c-set is actually scheduled in the frame (recall that by the nature of the problem, in the constructed frame each of the c-sets is used at most once). Thus, objective (3a) minimises the total frame length, i.e., the number of c-sets from family C that are actually required to be used. Note that due to minimisation in (3a), variables $T_{c}$ can be formally assumed to be continuous and unconstrained in sign, as in any optimal solution of (3) they will be binary anyway, and equal to 0 when a c-set *c* is not used, i.e., when $h_{vc}=0$ for all *v* in W(c).

The final (not counting the variable-range constraints (3e) and (3f)) is the energy constraint (3d); it imposes an upper bound on the energy consumed by the nodes broadcasting in the frame. Note that for Δ=0, the energy constraint assures that each node in B broadcasts its packet exactly once (which implies that U(c,v)⊇R(v) for $h_{vc}=1$) and in effect the minimal energy consumption (which is equal to |B|) is ensured. On the other hand, when Δ is a large number (in this case we write Δ=∞), the energy consumption is not taken into account and constraint (3d) can be deleted from the formulation. In this case, optimal solutions to FMP (C,∞) provide the shortest possible frame (for a given subfamily C). In the following, the minimum frame length resulting from FMP (C,Δ) will be denoted by T(C,Δ). In particular, T(C,0) will denote the minimum frame length when the minimum energy usage per frame is assumed, and T(C,∞) the absolute minimum of the frame length when energy consumption is not limited (both for a given C).

To clarify, the lower bound on the energy usage, equal to the number of broadcasting nodes |B|, is achieved when for each node v∈B the packet containing the data aggregated in *v* is broadcast only once, i.e., it is delivered to all nodes in R(v) in one time slot. This, as already discussed in Section 3, can be achieved using c-sets with only one broadcasting node each.

Note that the case with node-dependent broadcasting energy can be easily captured by substituting constraint (3d) with

(4) $$\sum_{v\in B}G\left(v\right)\sum_{c\in C(v)}h_{vc}\leq \sum_{v\in B}G\left(v\right)+\Delta ,$$

where G(v) is the broadcasting energy at node *v*, and $\sum_{v\in B}G\left(v\right)$ is the corresponding lower bound on energy usage. Note also that the energy spent on aggregation and packet reception is not considered in FMP, as the scheduling of packet broadcasts does not affect it.

Formulation (3) is non-compact as it assumes a fixed predefined list C of c-sets selected from the list $\hat{C}$ of all c-sets, and the size of the latter list is exponential with the size of the network graph. Certainly, in general a given list C will not provide an optimal solution when using FMP (C). This is guaranteed only when the full list $\hat{C}$ of c-sets, or a properly selected subset of $\hat{C}$, is used as the parameter to FMP. As for networks of practical sizes it is not feasible to pre-generate all possible c-sets and use them in FMP, generating a list C of reasonable size that guarantees an optimal solution of FMP is a critical issue that is addressed later in Section 5.

Finally, observe that the optimal frame composition $T_{c}^{*},\;c\in C,$ is capable of delivering all packets generated in the consecutive measurement cycles to their destinations with a finite delay when its length $T^{*}$ is less than or equal to the interval between measurements. The delay arises because the packet cannot be broadcast before its node v∈B collects (and aggregates) all the packets from all the nodes w∈B such that v∈R(w). Thus, the delay in delivering a packet from *o* to *d* over path P(o,d) will depend on the sequence according to which the c-sets actually used in the frame are assigned to the frame time slots. Minimising the number of frames required to deliver all packets in a given set of measurements is an issue that is out of scope of this paper (see in Appendix B of [3] and in [32]).

A given measurement may traverse one hop per frame, and thus in general requires multiple frames to be delivered to its destination(s). However, after an initial warm-up period, one full set of measurements can be delivered in each frame: each aggregator node collects, over the course of one frame, all measurements it is supposed to aggregate, and then can broadcast them, possibly in the subsequent frame. Once the pipeline of measurements traversing through the network is full, that is, the first set of measurements have reached their destinations, subsequent frames each deliver one new set of measurements. Therefore, minimisation of the frame length is equivalent to maximisation of the end-to-end throughput of the network.

## 5. Solution Algorithm

In order to solve FMP, we will first solve its linear relaxation by *column generation* (based on the so called pricing problem, see in [33,34]), and then solve the MIP version of FMP for the resulting family of c-sets. The linear relaxation (LR) of (3) will be called the *master problem* and denoted by MP(C,Δ) (the notation introduced in this section is listed in Table 3). MP(C,Δ) is a linear program (LP) obtained from (3) by assuming $h_{vc}\in R_{+}$ instead of $h_{vc}\in B$ (v∈B,c∈C(v)). Note that it is not necessary to assume $h_{vu}\leq 1$, as in any optimal solution of LR this constraint will be satisfied.

The *dual* to MP(C,Δ) (for the notion of the problem dual to an LP see in [33]) is as follows.

(5a)maxW=∑v∈B∑u∈R(v)λvu−(|B|+Δ)θ(5b)∑w∈W(c)πcw=1,c∈C(5c)∑u∈U(c,v)∩R(v)λvu≤πcv+θ,c∈C,v∈W(c)(5d)θ∈R+(5e)λvu∈R+,v∈B,u∈R(v);(5f)πcw∈R+,c∈C,w∈W(c).

It is worthwhile to note here, that for Δ=∞, i.e., in the case when the energy constraint (3d) is deleted from MP(C,Δ), the above dual formulation becomes simpler because then the dual variable θ, along with the terms involving θ in (5a) and (5c), disappear.

Let $\lambda^{*}={\left(\lambda_{vu}^{*}\right)}_{v\in B,u\in R(v)}$, $\pi^{*}={\left(\pi_{cw}^{*}\right)}_{c\in C,w\in W(c)}$ and $\theta^{*}$ specify an optimal solution of the dual (5) considered for a given family of c-sets C. Essentially, the *pricing problem* (PP) implied by the above dual consists in finding a c-set *c* in $\hat{C}\backslash C$ (if any) for which the constraints defining the dual polytope (considered for the family C∪{c}) projected onto the (θ,λ)-space (i.e., onto $R_{+}^{1+\sum_{v\in B}\left|R\left(v\right)\right|}$) are *most violated* by $\lambda^{*}$ and $\theta^{*}$. In fact, PP can solved by means of a max-min problem that finds a c-set *c* in $\hat{C}$ which maximises the expression

(6) $$P\left(c\right)=\min_{\pi \geq 0:\;\sum_{w\in W(c)}\pi_{w}=1}Q\left(\pi ;c\right),$$

where the quantity Q(π;c), corresponding to constraints (5c), is defined as

(7) $$\sum_{v\in W(c)}\max\left\{0,\sum_{u\in U(c,v)\cap R(v)}\lambda_{vu}^{*}-\theta^{*}-\pi_{v}\right\}.$$

Now suppose that the maximum (6) of PP is positive: $P^{*}=P\left(c^{*}\right)>0$, where the c-set $c^{*}$ is an optimal solution of PP. Then, adding $c^{*}$ to the dual problem (that is, adding the dual variables $\pi_{c^{*}w},w\in W\left(c^{*}\right),$ and the appropriate constraints (5b) and (5c)) will cut off some parts of the dual polytope, and in particular make $\lambda^{*}$ and $\theta^{*}$ infeasible. Thus, introducing $c^{*}$ will in general decrease the maximum $W^{*}$ of the dual objective function (5a). As $W^{*}$ is equal to the minimum $T^{*}$ of the primal objective function (3a) (see in [33]), it follows that (potentially) the modified master MP($C\cup \{c^{*}\},\Delta$) has a smaller minimum than MP(C,Δ).

This observation leads to an iterative c-set generation process: the so-obtained $c^{*}$ is added to C ($C:=C\cup \{c^{*}\}$), and the iteration is repeated starting with re-solving MP(C,Δ) for the updated family of c-sets. Finally, when $P^{*}$ becomes equal to 0, the process is terminated, as this means that no more c-sets are required in C to achieve the minimum of MP($\hat{C},\Delta$) (the minimum of MP(C,Δ) for the current C is equal to the minimum of MP($\hat{C},\Delta$)). Finally, when the c-set generation process terminates, the original MIP version of FMP in formulation (3) is solved by a MIP solver for the so-obtained family C.

Note that after solving MP(C,Δ), the LP solver delivers the optimal dual variables, apart from the optimal primal variables. Moreover, in the next iteration the optimal basis of MP(C,Δ) can be used as the initial basis for MP($C\cup \{c^{*}\},\Delta$) (a so called *warm start*).

To perform the above described c-set generation process we need to treat the pricing problem (6) by means of a MIP formulation. Obtaining such a formulation is not straightforward, and to achieve this we follow the method described in [3]. First, we formulate the task of computing P(c) (for a given c-set $c\in \hat{C}$ as an LP formulation of the minimisation type (this is easy) and then take its dual, which uses continuous variables *f* (a scalar) and $g_{v},\;v\in B$. Finally, we merge this dual with the c-set defining formulation (1) to obtain the following pricing problem formulation in variables $Y_{vu},v\in V,u\in R\left(v\right)$, $X_{v},v\in B$, *f* and $g_{v},v\in B$:

(8a)maxP=−f+∑v∈B(∑u∈R(v)λvu*Yvu)gv−θ*gvc-set defining constraints on Xv,Yvu:(1a)−(1f)(8b)gv≤f,v∈B(8c)gv≤1,v∈B(8d)Yvu∈B,v∈B,u∈R(v)(8e)Xv∈B,v∈B(8f)f∈R(8g)gv∈R+,v∈B.

As discussed in [3], problem (8) is NP-hard. Nonetheless, as illustrated in Section 6, solving (8) using a MIP solver is quite effective.

The optimal c-set $c^{*}$ is defined by an optimal solution of (8) through

(9) $$W\left(c^{*}\right):=\left\{v\in B:\;X_{v}^{*}=1\right\},\;U\left(c^{*},v\right):=\left\{u\in V:\;Y_{vu}^{*}=1\right\},\;v\in W\left(c^{*}\right).$$

Note that in order to convert formulation (8) to a proper MIP, the products of variables $Y_{vu}\cdot g_{v}$ in (8a) need to be linearised (in the standard way, see in [3]). Clearly, in the case when the energy constraint (3d) is deleted from the master problem, the term containing $\theta^{*}$ in (8a) disappears.

In summary, the overall (*price-and-branch*, see in [35]) algorithm used to solve the considered optimisation problem is as follows (Algorithm 1).
**Algorithm 1:** FMP algorithm
       **Step 0:** Form the initial c-set family C:=C(B) (where C(B)={c(v):v∈B}).
       **Step 1:** Solve the master MP(C,Δ), i.e., the linear relaxation of FMP(C,Δ) formulated in (3), to obtain the optimal dual variables $\lambda^{*}={\left(\lambda_{vu}^{*}\right)}_{v\in B,u\in R(v)}$ and $\theta^{*}$.
       **Step 2:** Solve the pricing problem (8) for parameters $\lambda^{*}$ and $\theta^{*}$. If the maximum of the objective function *P* is strictly positive, then $C:=C\cup \{c^{*}\}$ (where $c^{*}$ is defined in (9)) and go to Step 1.
       **Step 3:** Solve the original MIP version of FMP(C,Δ) and stop.

The minimum frame length obtained with the FMP algorithm for a given value of parameter Δ will be denoted by T(Δ). Additionally, the frame length obtained for the original MIP assuming the initial c-set family C(B) will be denoted by T(C(B)). As we already know (see Section 3.4), in this case, formulation (3) has a (trivial) unique optimal solution $h_{vc(v)}^{*}=1,v\in B$, with the frame length equal to |B|. This solution is valid for all Δ≥0 and therefore is optimal as far as energy consumption is concerned. Thus, T(C(B))=|B| (=T(C(B),Δ) for all Δ≥0).

## 6. Numerical Study

In order to investigate the trade-offs between energy minimisation and throughput maximisation, we conducted a numerical study in which we implemented formulations (3)–(8) as problems in the AMPL modelling language and solved them with the CPLEX optimisation solver using the approach described in Section 5. All problem instances were solved on an Intel Core i7-3770K CPU (3.5 GHz) with 8 virtual cores (4 cores with 2 threads each) and 8 GB RAM.

As input data, we used the routing configurations optimised (and described) in [1], which consist of wireless mesh network topologies of varying sizes from 10 to 40 nodes, together with routing, aggregation and dissemination solutions for total energy minimisation and for min-max energy optimisation. (For min-max energy optimisation, only network configurations up to 35 nodes in size were available.) Note that the quantities characterizing radio transmission used to calculate the parameters appearing in the c-set definition (1), i.e., p(v,w),η,γ, are consistent with those used in [1] (node transmission power 20 mW, path loss proportional to distance with exponent 4, noise power −81 dBm, SINR threshold 8 dB).

All results in the following are shown with 95% confidence intervals, taken across the randomly generated (see in [1]) set of routing configurations for each network size. While in some machine-to-machine communication scenarios larger network sizes may occur, the energy minimisation problems from [1] are computationally expensive to solve, so in the current work we limit our study to the existing datasets.

For each problem instance, we applied the FMP algorithm to find the minimum frame length without energy margin (Δ=0, minimum energy consumption assumed) and with infinite energy margin (Δ=∞, energy constraint (3d) skipped), giving, on the one hand, the minimum frame achievable while keeping the energy used to the minimum, and on the other hand, the minimal frame achievable for the given routing configuration disregarding energy usage. As indicated in Step 0 of the FMP algorithm, to initialise the c-set generation algorithm, we started with family C(B) of c-sets each consisting of one transmitting node with its immediate neighbours as receivers. As already mentioned, this family of c-sets ensures the feasibility of formulation (3) already without energy margin.

**Figure 2:**
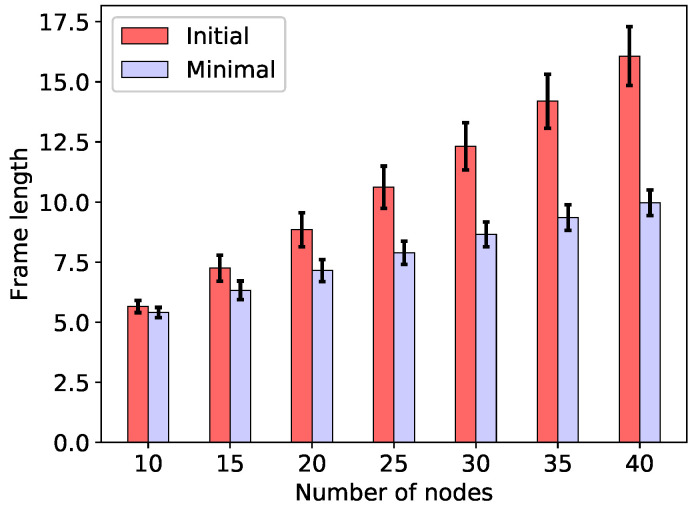
Initial (T(C(B))) and minimal (T(∞)) frame lengths for minimum total energy configurations.

**Figure 3:**
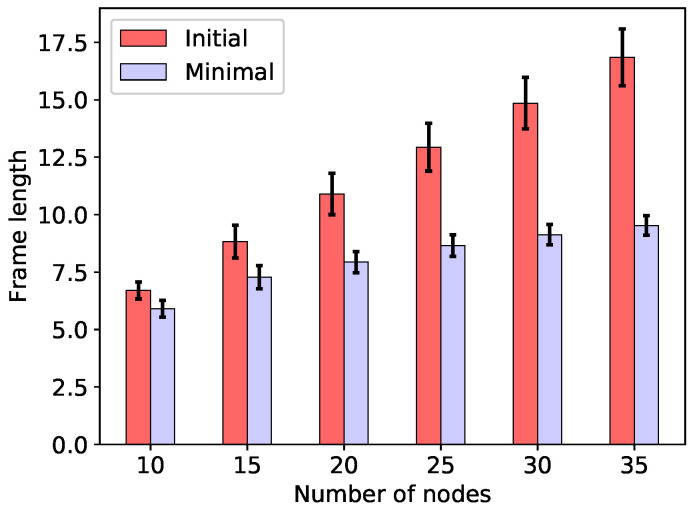
Initial (T(C(B))) and minimal (T(∞)) frame lengths for min-max energy configurations.

The initial frame lengths T(C(B)) (equal to |B|) obtained using only the initial c-set family C(B), along with the minimum frame lengths T(∞) obtained by means of the FMP algorithm when the energy constraint (3) is skipped, are shown in Figure 2 for the minimal total energy configurations, and in Figure 3 for the min-max energy configurations. As can be seen in the figures, not only is the frame substantially reduced through c-set generation and frame optimisation, but the minimal frame length also grows more slowly with the size of the network than does the initial frame length. Finally, note that the two considered frame lengths, give, respectively, the upper and the lower bound on the minimum frame length necessary to ensure minimum energy consumption T(0) (which is not considered in Figure 2 and Figure 3): T(C(B))≥T(0)≥T(∞).

Now let us proceed to the main issue, i.e., the trade-off between the frame length and energy consumption minimisation. In almost all problem instances tested, it was possible to obtain minimum frame while also keeping energy at the minimum, showing that minimising the frame length provides only additional benefits, without negatively impacting energy usage. In fact, using a shorter frame will be directly beneficial to energy usage if nodes are able to sleep between transmissions.

**Figure 4:**
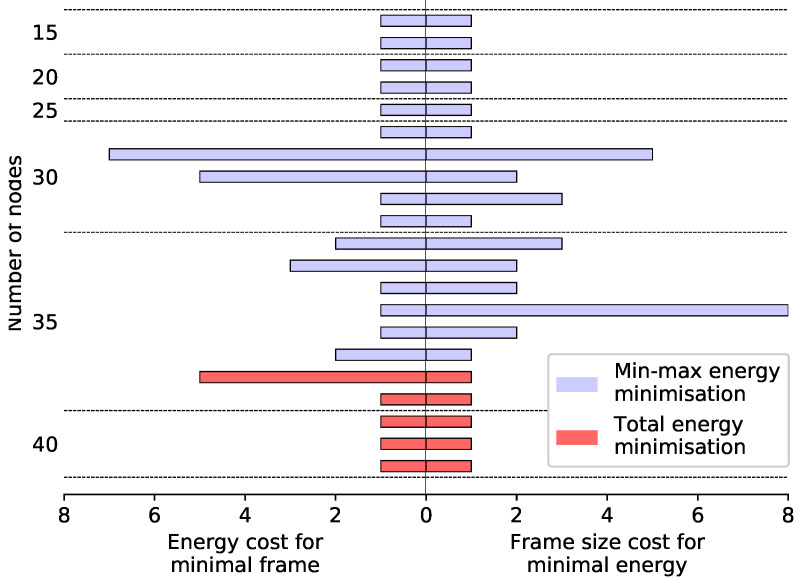
Trade-offs between energy and throughput minimisation. For each of the 21 cases where the minimal-energy frame was larger than the minimal-length frame, the energy margin Δ necessary to achieve the minimal frame length T(∞) is shown on the left, while the frame length increase T(0)−T(∞) necessary to achieve the minimal energy is shown on the right. Cases shown in blue were produced from the min-max energy minimisation dataset, while those shown in red were from the total energy minimisation dataset.

Thus, in almost all cases the minimum frame length and minimum energy can be achieved simultaneously; however, in 21 of the in total 260 problem instances we tested there was a trade-off between the two. For these cases, we relaxed the energy constraint (3d) by successively increasing energy margin Δ on the right-hand side to allow the energy usage to increase until T(Δ) goes down to the absolute minimum frame length T(∞), the results of which are shown in Figure 4 and Table 4. In most cases, even when there is a trade-off between energy usage and throughput, it is small—only one extra time slot or one extra transmission is needed to optimise the other objective. However, anomalous cases where the costs are higher are possible.

It should also be noted that most trade-off cases occurred at larger network sizes, and there were many more for min-max energy configurations than for minimal total energy configurations, even though our datasets included larger networks for total energy minimisation. This is because the min-max configurations tend to balance the energy load more evenly amongst the nodes, resulting in transmissions that are spread out throughout the network, rather than concentrated along one or a few paths. As transmissions become more dispersed, it is more likely that some nodes will need to transmit more than once in order to achieve the minimum frame length. (Note that in general, sending a packet from a given node to its neighbours using more than one broadcast allows for increasing the number of broadcasting nodes in the slots in which the packet in question is broadcast.) Such cases are thus more common in larger networks and in min-max configurations—for min-max energy at the largest network size of 35 nodes, more than one-quarter of the instances tested showed some trade-off between the two objectives. Our models and solution approach provide a means to manage these performance trade-offs, with the energy margin added to constraint (3d) controlling how much extra energy it is permissible to use in order to increase the throughput, and the minimum frame length giving a bound on the throughput that can be achieved in this way.

**Figure 5:**
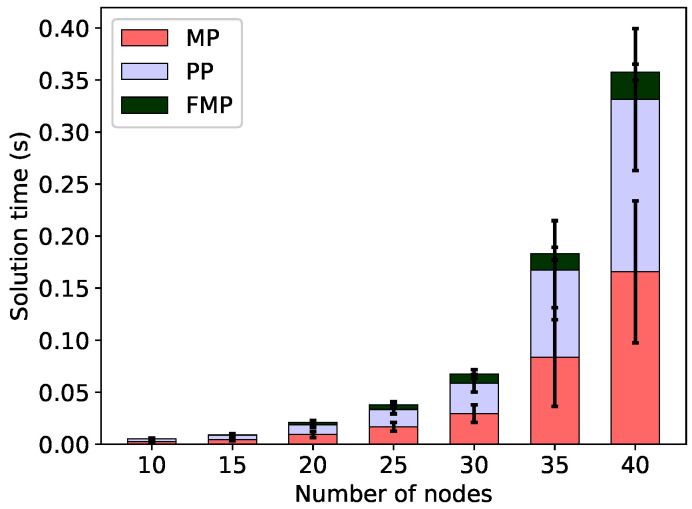
Solution times to solve the linear master problem (MP), pricing problem (PP) and the final integer problem (FMP) for the minimal frame; no energy constraint, minimum total energy configurations.

**Figure 6:**
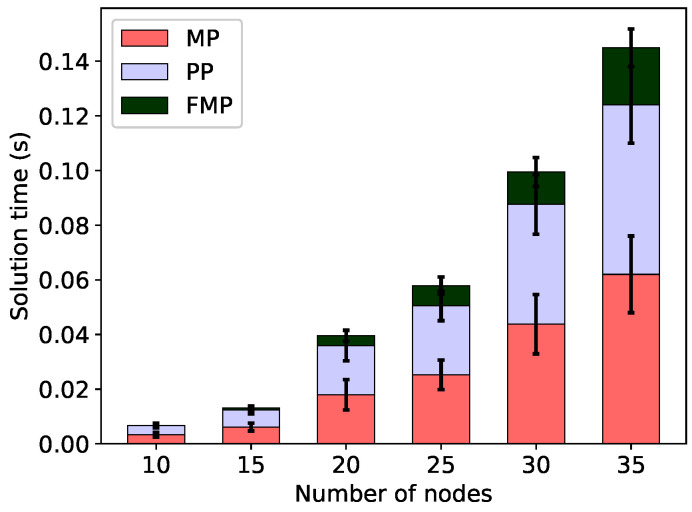
Solution times to solve the linear master problem (MP), pricing problem (PP) and the final integer problem (FMP) for the minimal frame; no energy constraint, min-max energy configurations.

As far as the computational efficiency is concerned, we observe that minimising the frame length by means of the FMP algorithm is efficient. This is illustrated in Figure 5 for minimal total energy configurations and in Figure 6 for min-max energy configurations. Both figures show the time required to find the minimum frame length, broken down into the time spent in the master problem, pricing problem and final integer problem, without the energy constraint (Δ=∞). For all 260 problem instances, these times are very short, less than 0.5 s.

**Figure 7:**
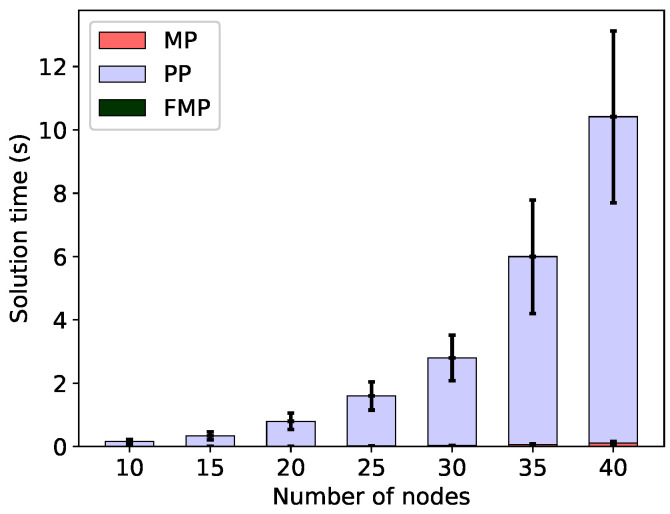
Solution times to solve the linear master problem (MP), pricing problem (PP) and the final integer problem (FMP) for the minimal frame; energy constrained to be minimal (energy margin Δ=0), minimum total energy configurations.

**Figure 8:**
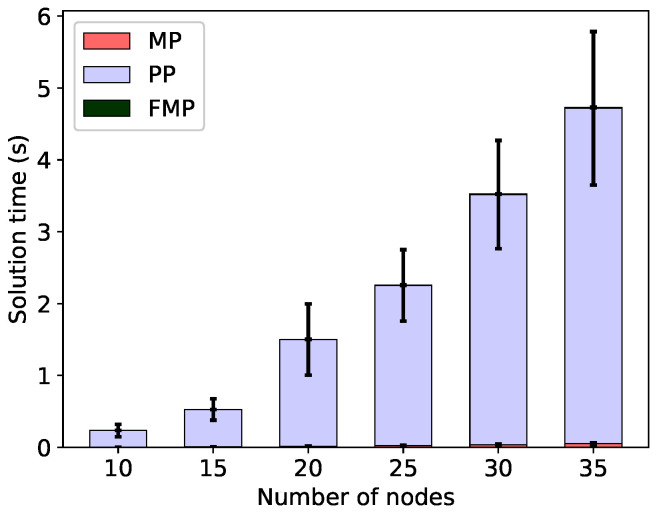
Solution times to solve the linear master problem (MP), pricing problem (PP) and the final integer problem (FMP) for the minimal frame; energy constrained to be minimal (energy margin Δ=0), min-max energy configurations.

However, when the minimal energy usage (Δ=0) is assumed, the FMP algorithm solution time increases. This is shown in the corresponding Figure 7 and Figure 8. Although the total solution time is still quite reasonable—of the order of seconds—it is much longer than the sub-second times observed without the energy constraint. With constrained energy, the solution time is also completely dominated by the time spent in the pricing problem; in fact the time spent in the linear master problem (the next largest component) is actually shorter than without the energy constraint. This shows that although it is usually possible to find c-sets that achieve the minimum frame at the minimum energy, this is much more difficult than only finding c-sets that achieve the minimum frame.

To end this section, let us note that networks of larger size, with |B| of the order of hundreds, say, have not been treated in this study mainly because the exact optimisation model of the work in [1], used for preparing the routing configurations, is not capable of dealing directly with such cases. For that, a heuristic algorithm would have to be devised, while the proposed problem formulation and the exact solution algorithms could still be used for benchmarking the developed heuristics on smaller networks. Moreover, a heuristic algorithm could use the proposed formulations as its building blocks. For example, if the network graph was decomposed by hierarchically partitioning the set of sensor and aggregator nodes, the formulations in [1] could still be used to solve the routing configuration problem for each of the resulting node subsets.

## 7. Concluding Remarks

In this paper, we have provided mixed-integer programming formulations and an effective solution algorithm to optimise the throughput in energy-efficient wireless mesh networks performing machine-to-machine communication, in which sensor measurements are aggregated and then disseminated via multicast transmission to multiple actuator nodes. Further, we have conducted a numerical study to test the performance of our models. For all network sizes tested, solution times were of the order of seconds, which makes them easily practical for implementation in real-world scenarios; for machine-to-machine communication, traffic is typically periodic, meaning that the optimisation problem needs only be solved once and then used for the duration of the given application’s run time.

Our results show that in almost all cases, throughput can be maximised without increasing the energy usage above the minimum, both for minimal total energy and for min-max energy usage, in which energy costs are shared more fairly amongst the nodes in the network. Using the approach we provide here, the throughput can be almost doubled when compared with the base approach of serialising transmissions in order to ensure the minimal energy usage is feasible to achieve. Aside from the direct benefit of improved throughput, this also increases nodes’ sleep times, and thus further improves their energy usage, as once all transmissions have been made for a given set of sensor measurements, nodes may be switched to sleep mode. Even in the few cases where the maximum throughput can only be achieved by incurring some energy cost, these costs are in most cases low. For such cases, our models provide a bound on the throughput that can be achieved when allowing higher energy usage, as well as a means to control the balance between throughput and energy usage.

As far as the extension of the presented work is concerned, our main goal for the near future is to devise efficient heuristics and decomposition methods (characterised at the end of Section 6) that will be effective for large networks.

Finally, we note that the performance considerations described in the numerical study, illustrating the (reasonable) trade-off between throughput and energy, are non-obvious, and they required the precise and efficient FMP algorithm introduced in this paper to be found.

## Figures and Tables

**Table 1:** General notation.

V	set of nodes (vertices) in the network
O	set of origin nodes
N	set of aggregator nodes
D	set of destination nodes
|A|	number of elements (size) of an arbitrary set A
*K*	number of packets that need to be collected
O(d)	set of origin nodes whose measurements should reach destination d∈D
P(o,d)	path from origin o∈O to destination d∈D
V(o,d)	set of transit nodes in path P(o,d)
B	set of broadcasting nodes
R(v)	set of nodes that receive the packet sent from node v∈V
$\hat{C}$	family of all c-sets
W(c)	set of simultaneously broadcasting nodes in c-set $c\in \hat{C}$
U(c,v)	set of nodes in c-set $c\in \hat{C}$ receiving the signal from v∈W(c)
c(v)	c-set with W(c(v))={v} and U(c(v),v)=R(v)
C(B)	family of all c-sets c(v) (C(B)={c(v):v∈B})
$X_{v}$	binary decision variable to determine if node v∈B will broadcast in a given c-set
$Y_{vu}$	binary decision variable to determine if node u∈V receives from v∈B in a given c-set
p(v,w)	transmission power received at node w∈V from node v∈V
η	noise power
γ	SINR threshold
T	set of time slots in each frame, T={1,2,…,T}
c(t)	c-set to be applied in time slot t∈T
B, R, $R_{+}$	sets of binary ({0,1}), real, and non-negative real numbers, respectively

**Table 2:** Frame minimisation problem—notation.

FMP(C,Δ)	frame minimisation problem (for a given subfamily of c-sets C)
C	subfamily of $\hat{C}$
Δ	allowable energy margin for node broadcasts in the frame (Δ≥0)
T(C,Δ)	minimum frame length for given C and Δ (solution of FMP(C,Δ))
T(C,0)	minimum frame length for given C for minimum energy (solution of FMP(C,0))
T(C,∞)	absolute minimum frame length for given C (solution of FMP(C,∞))
C(v)	set of c-sets in C in which node *v* is broadcasting (C(v):={c∈C:v∈W(c)})
C(v,u)	set of c-sets in C(v) in which node *u* is receiving from node *v*
	(C(v,u):={c∈C(v):u∈U(c,v)})
$T_{c}$	decision variable: number of times c-set c∈C is scheduled during the frame
$h_{vc}$	decision variable: equals 1 iff node v∈B broadcasts in c-set c∈C during the frame

**Table 3:** Master problem, dual problem and pricing problem—notation.

MP(C,Δ)	master problem: linear relaxation of FMP (for a given subfamily of c-sets C)
λ,π,θ	dual variables: $\lambda_{vu},v\in B,u\in R\left(v\right);\;\pi_{cw},\;c\in C,w\in W\left(c\right);\;\theta$
$\lambda^{*},\pi^{*},\theta^{*}$	optimal dual variables
$P^{*}=P\left(c^{*}\right)$	optimal value of the pricing problem achieved for c-set $c^{*}$
T(C(B))	minimum frame length for family C(B) (T(C(B))=|B|)

**Table 4:** Trade-off between energy and frame length.

Case	T1	M1	M2	M3	M4	M5	M6	M7	M8
number of nodes	35	30	30	30	35	35	35	35	35
T(0): minimum frame length with energy margin 0	20	19	14	10	13	14	10	11	11
T(∞): minimum frame length (with no energy margin)	15	12	9	9	11	11	9	10	9
T(0)−T(∞): increase in frame length with energy margin 0	5	7	5	1	2	3	1	1	2
Δ: energy margin necessary to reach minimum frame	1	5	2	3	3	2	2	8	1

Cases where energy margin Δ greater than zero was required to achieve the minimal frame length. Cases labelled with “T” indicate total energy minimisation, while those with “M” indicate min-max energy minimisation. Twelve cases are omitted from the table (see the text for details).
